# Left atrial strain - an early marker of left ventricular diastolic dysfunction in patients with hypertension and paroxysmal atrial fibrillation

**DOI:** 10.1186/s12947-018-0147-6

**Published:** 2018-10-31

**Authors:** Jonas Jarasunas, Audrius Aidietis, Sigita Aidietiene

**Affiliations:** Clinic of Cardiac and Vascular Diseases, Institute of Clinical Medicine, Faculty of Medicine, Vilnius University, Universiteto g. 3, LT-01513 Vilnius, Lithuania

**Keywords:** Left atrial strain, Diastolic dysfunction, Arterial hypertension, Atrial fibrillation

## Abstract

**Background:**

2D strain imaging of the left atrium (LA) is a new echocardiographic method which allows us to determine contractile, conduit and reservoir functions separately. This method is particularly useful when changes are subtle and not easily determined by traditional parameters, as it is in arterial hypertension and atrial fibrillation (AF). The aims of our study were: to determine LA contractile, conduit and reservoir function by 2D strain imaging in patients with mild arterial hypertension and paroxysmal AF; to assess LA contractile, conduit and reservoir functions’ relation with LV diastolic dysfunction (DD) parameters.

**Methods:**

LA contractile, conduit and reservoir functions together with echocardiographic signs of LV DD were assessed in 63 patients with arterial hypertension and paroxysmal AF. Patients were grouped according to number of signs showing LV DD (annular e’ velocity: septal e’ < 7 cm/s, lateral e’ < 10 cm/s, average E/e’ ratio > 14, LA volume index > 34 ml/m^2^, peak tricuspid regurgitation velocity > 2.8 m/s) present. Number of patients with 0 signs – 17, 1 sign – 26, 2 signs – 19. Contractile, conduit and reservoir functions were compared between the groups.

**Results:**

Mean contractile, conduit and reservoir strains in all the patients were − 14.14 (± 5.83) %, 15.98 (± 4.85) % and 31.03 (± 7.64) % respectively. Contractile strain did not differ between the groups. Conduit strain was higher in patients with 0 signs compared with other groups (*p* = 0.016 vs 1 sign of LV DD and *p* = 0.001 vs 2 signs of LV DD). Reservoir strain was higher in patients with 0 signs compared with other groups (*p* = 0.014 vs 1 sign of LV DD and *p* < 0.001 vs 2 signs of LV DD).

**Conclusions:**

The patients with paroxysmal AF and primary arterial hypertension have decreased reservoir, conduit and pump LA functions even in the absence of echocardiographic signs of LV DD. With increasing number of parameters showing LV DD, LA conduit and reservoir functions decrease while contractile does not change. LA conduit and reservoir functions decrease earlier than the diagnosis of LV DD can be established according to the guidelines in patients with primary arterial hypertension and AF.

## Background

Traditionally the greatest attention during a routine echocardiography is paid to the function of the ventricles and assessment of the atria is limited to measuring the dimensions and volumes of the chambers. Though assessing ventricular function is essential, there is robust data that atrial function is also important and can improve our decision making by determining the risk of cardiovascular events in various conditions [1]. Hypertension is the most common predisposing factor for left ventricular (LV) diastolic dysfunction (DD), which leads to increased left atrial (LA) pressure, its enlargement and fibrosis as well as other proarrhythmic pathological effects on atrial structure and function [2, 3]. These changes cause various cardiac arrhythmias, most commonly atrial fibrillation (AF), an arrhythmia that carries a substantial risk of embolic events. Hypertension and even high-normal blood pressure is a risk factor for developing AF and recent guidelines for the management of arterial hypertension clearly state that AF should be considered a manifestation of hypertensive heart disease [4]. LA function might also be linked to the cardioembolic risk profile in patients with AF and can even provide incremental value for embolism risk stratification over CHA2DS2-VASc score [5, 6]. Recently announced EACVI AFib Echo Europe Registry for assessing relationships of echocardiographic parameters with clinical thrombo-embolic and bleeding risk profile in non-valvular AF aims to determine echocardiographic parameters stratifying prognosis and improving management in categories of AF patients. In this regard LA parameters are among the most promising ones [7].

There are many well established and validated methods to assess left and right ventricular function but the ones for assessing atrial function are lacking. Speckle tracking echocardiography has proven to be useful and applicable not only in the assessment of LV wall motion abnormalities but also in the assessment of LA function. Though the method is more and more studied it is still not widely used in daily clinical practice primarily because there are still some methodological and standardization issues which need to be addressed. The question of normal values is also still valid, though metanalysis by Faraz Pathan et al. was a real step forward in determining normal ranges [8].

One of the most promising areas the method can be used in is hypertension where the LA and LV dysfunctions occuring early in the course of disease can be subtle and not easily determined by traditional echocardiographic parameters [9]. 2D strain parameters of the LA can help to detect increased filling pressures and DD of the LV earlier [10], and, which is very important, antihypertensive treatment can reverse these changes [11].

The aims of our study were: a) to determine LA contractile, conduit and reservoir function by 2D strain imaging in patients with mild arterial hypertension and paroxysmal atrial fibrillation; b) to assess LA contractile, conduit and reservoir functions’ relation with LV filling pressure parameters recommended by the American Society of Echocardiography and the European Association of Cardiovascular Imaging [12].

## Methods

We assessed 63 patients aged 18–80 with I or II grade primary arterial hypertension and at least one ECG confirmed episode of paroxysmal AF within last year. Only the patients that were in sinus rhythm at the time of investigation were included in the study. Patients with other known causes of AF such as heart failure, coronary heart disease, prior heart surgery, structural heart disease, reduced LV ejection fraction, thyroid dysfunction (assessed by thyroid-stimulating hormone concentration) or renal failure with glomerular filtration rate < 60 ml/min were excluded from the study. Only the ones with hypertension as a possible causative factor for AF were included in the study.

Physical examination, including weight and height was performed. All patients underwent ambulatory blood pressure monitoring, which was carried out according to European Society of Hypertension guidelines [13]. A Meditech card(X)plore monitor and CardioVisions 1.23.0 software were used. The measurements were taken in 20-min intervals during the day and in 40-min intervals during the night. The patients who did not meet the 70% successful measurement criterion were excluded from the analysis.

All the patients had an ECG and sonography of the heart done. Only the ones with acceptable ultrasound image quality were included in the final analysis. A GE Vivid E9 system was used for ultrasound imaging in our study. Routine sonographic examination of the heart was performed as described in the American and European Society of Echocardiography guidelines and their update [14–16] with a cardiac probe M5S-D.

The thicknesses of the interventricular septal and the inferolateral walls as well as LV end-diastolic and end-systolic diameters were obtained from the parasternal short-axis view. LV mass (LVM) was calculated using linear method as recommended in the update Recommendations for cardiac chamber quantification by echocardiography in adults [16]. Cube formula was used:

$$ \mathsf{LVM}=\mathsf{0.8}\ \mathsf{x}\ \mathsf{1.04}\ \left[{\left(\mathsf{LVEDd}+\mathsf{PWDd}+\mathsf{IVSDd}\right)}^{\mathsf{3}}-{\mathsf{LVEDd}}^{\mathsf{3}}\right]+\mathsf{0.6}\ \mathsf{g}, $$

where LVEDd is LV end-diastolic internal diameter; PWDd, diastolic posterior wall thickness; and IVSTd, diastolic interventricular septal thickness. To determine LV hypertrophy LVM was subsequently indexed to body surface area BSA (calculated using DuBois formula). Two waves (E and A) of mitral inflow velocity were recorded using pulsed wave Doppler from the apical 4 chamber view. The velocity waves (e’ and a’) of mitral annulus septal and lateral regions were recorded using tissue Doppler. When calculating E/e’ ratio, an average value of septal and lateral mitral annulus velocities was used.

LV and LA volumes were determined using the biplane disk summation technique from apical 4-chamber and 2-chamber views. LV end systolic and end diastolic volume was recorded, then LV ejection fraction was calculated using these measurements.

**Fig. 1:**
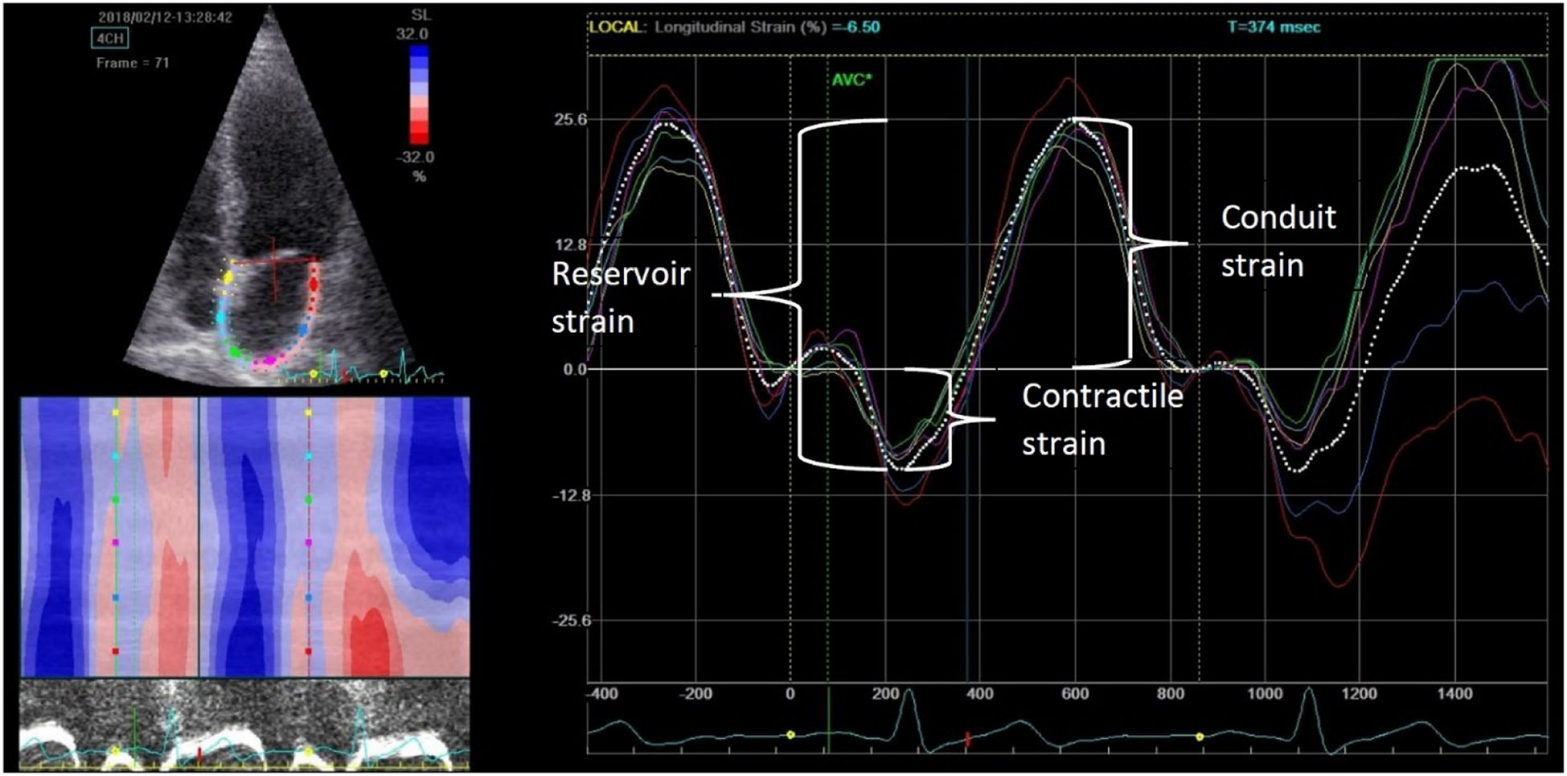
2D LA strain image from four-chamber apical view. Setting the starting point of strain analysis at the beginning of the p wave on the ECG allowed us to define first negative peak, first positive peak and the difference of these peaks which corresponded to atrial contractile strain, conduit strain and reservoir strain respectively

Global longitudinal 2D LA strain was analyzed by the speckle tracking technique using GE EchoPAC software. The images were acquired according to the recommendations given by expert consensus statement published in the European Journal of Echocardiography [17]. For analysis we used four-chamber and two chamber apical view images of LA carefully avoiding foreshortening. The focus was set to the level of mid-LA to optimize the image quality. Sector depth and width was adjusted to include as little as possible outside the region of interest. Three consecutive heart cycles were recorded during a single breath hold using a frame rate of > 80 frames/second for offline analysis. The endocardial border of LA was manually traced and a region of interest was manually adjusted to include the entire LA wall thickness. The software selected stable speckles within the LA wall and tracked these speckles frame-by-frame throughout the cardiac cycle. The entire LA tracking was then divided into 6 segments by the software and tracking quality for each segment was provided. If the tracking was not acceptable, endocardial borders were readjusted until better tracking was achieved. Then, we set the starting point of strain analysis as P-wave onset instead the software preset R-wave peak. The automated software then generated traces depicting the regional longitudinal strain for each segment and calculated global longitudinal strain. Using P wave onset as starting enabled us to define first negative peak, which occurred at maximal LA contraction and represented its contractile function (contractile strain), first positive peak, which occurred at mitral valve opening and represented LA conduit function (conduit strain), and the difference of these peaks, which represented reservoir function (reservoir strain). The values were averaged for all 12 LA segments - 6 in apical four chamber view and 6 in apical two chamber view. LA strain image from four-chamber apical view is shown in Fig. 1. Analogous measurements were performed from apical two-chamber views.

LV diastolic function and filing pressures were evaluated according to the American Society of Echocardiography and the European Association of Cardiovascular Imaging recommendations published in 2016 [12]. The patients were grouped as having none, one, two or three signs of LV DD, according to the guidelines. The variables for identifying LV DD and their cutoffs were annular e’ velocity: septal e’ < 7 cm/s, lateral e’ < 10 cm/s, average E/e’ ratio > 14, LA volume index > 34 ml/m^2^, peak tricuspid regurgitation velocity > 2.8 m/s.

The intra- and interobserver variability of contractile, conduit and reservoir LA strains was assessed in 20 randomly selected patients. Intraobserver variability was performed by the same echocardiographer blinded to previous measurements and interobserver variability was performed by a second experienced echocardiographer also blinded to previous measurements. The intraclass correlation coefficient together with the absolute difference divided by the mean of two measurements and given as a percentage were calculated for both intra- and interobserver variability.

For statistical analysis Microsoft Excel and SPSS Statistics 17.0 software was used. The mean values are presented ± standard deviation (SD) or 95% confidence intervals (CI). Shapiro-Wilk test was used to check if the distribution of the data was normal. The means were compared using ANOVA and Fisher’s Least Significant Difference test was used for post hoc analysis. Pearson’s correlation coefficient was used to test for correlation. *P* value of < 0.05 was considered significant.

## Results

Sixty-three patients who met inclusion/exclusion criteria, had acceptable ultrasound picture quality and signed informed consent were included in the final analysis. The patients’ demographic, physical examination and blood pressure data is presented in Table 1. Ultrasound of the heart data is presented in Table 2. Intraclass correlation coefficients (95% CI) for intraobserver variability of LA contractile, conduit and reservoir strains were 0.91 (0.79—0.96), 0.92 (0.81—0.97) and 0.94 (0.86—0.98) respectively. The absolute difference divided by the mean of two measurements for intraobserver variability of LA contractile, conduit and reservoir strains was 5.7%, 5.5% and 4.9% respectively. Intraclass correlation coefficients (95% CI) for interobserver variability of LA contractile, conduit and reservoir strains were 0.89 (0.75—0.96), 0.91 (0.79—0.96) and 0.93 (0.82—0.97) respectively. The absolute difference divided by the mean of two measurements for interobserver variability of LA contractile, conduit and reservoir strains was 8.6%, 6.0% and 5.5% respectively. 2D strain parameters of LA are shown in Table 3. Table 4 shows comparison of contractile, conduit and reservoir strain data of our study population with normal values of healthy individuals according to metanalysis by Faraz Pathan [8].

**Table 1:** Demographic, physical examination and blood pressure data of the study population

Variable	Study population (± SD)
Age	63.08 (±11.54)
Male	41%
Height	1.71 (±0.09) m
Weight	86.56 (±15.02) kg
Body mass index (BMI)	29.52 (±4.35) kg/m^2^
Body surface area (BSA)	1.98 (±0.2) m^2^
24 h average systolic blood pressure	128.94 (±10.75) mm Hg
24 h average diastolic blood pressure	74.42 (±8.26) mm Hg
Smokers	7.9%
Number of different antihypertensive agents taken daily	1.57 (±1.15)

**Table 2:** Ultrasound data of the study population

Variable	Study population (± SD)
LV ejection fraction	61.48 (± 5.04) %
LVEDd	5.08 (± 0.5) cm
LV end-diastolic volume	95.16 (± 24.64) ml
IVSDd	1.05 (± 0.12) cm
PWDd	0.91 (± 0.1) cm
Indexed LV mass	92.76 (± 19.7) g/m^2^
LA diameter	40.86 (± 6.40) mm
LA volume	70.98 (± 20.12) ml
Indexed LA volume (LAVI)	35.80 (± 9.64) ml/m^2^
E/A ratio	1.11 (± 0.47)
Average septal e’	7.92 (± 0.57) cm/s
Average lateral e’	9.87 (± 2.87) cm/s
E/e’ ratio	8.68 (± 2.89)
IVRT	94.31 (± 21.78) ms

**Table 3:** LA strain data of the study population

Variable	Study population (± SD)
Mean contractile strain	−14.14 (± 5.83) %
4CH contractile strain	−13.90 (± 4.51) %
2CH contractile strain	−14.39 (± 9.19) %
Mean conduit strain	15.98 (± 4.85) %
4CH conduit strain	14.99 (± 4.63) %
2CH conduit strain	16.97 (± 5.78) %
Mean reservoir strain	31.03 (± 7.64) %
4CH reservoir strain	28.89 (± 7.30) %
2CH reservoir strain	33.17 (± 9.28) %

**Table 4:** LA strain data compared with normal values in healthy individuals

Variable	Study population (95% CI)	Normal values according to metanalysis (95% CI) [8]
Mean contractile strain	−14.14 (−15.61–-12.67) %	17.4 (16.0–19.0) %
Mean conduit strain	15.98 (14.76–17.20) %	23.0 (20.7–25.2) %
Mean reservoir strain	31.03 (29.11–32.96) %	39.4% (38.0–40.8) %

Contractile, conduit and reservoir strains in patients with mild hypertension and paroxysmal AF are lower compared to normal population

**Fig. 2:**
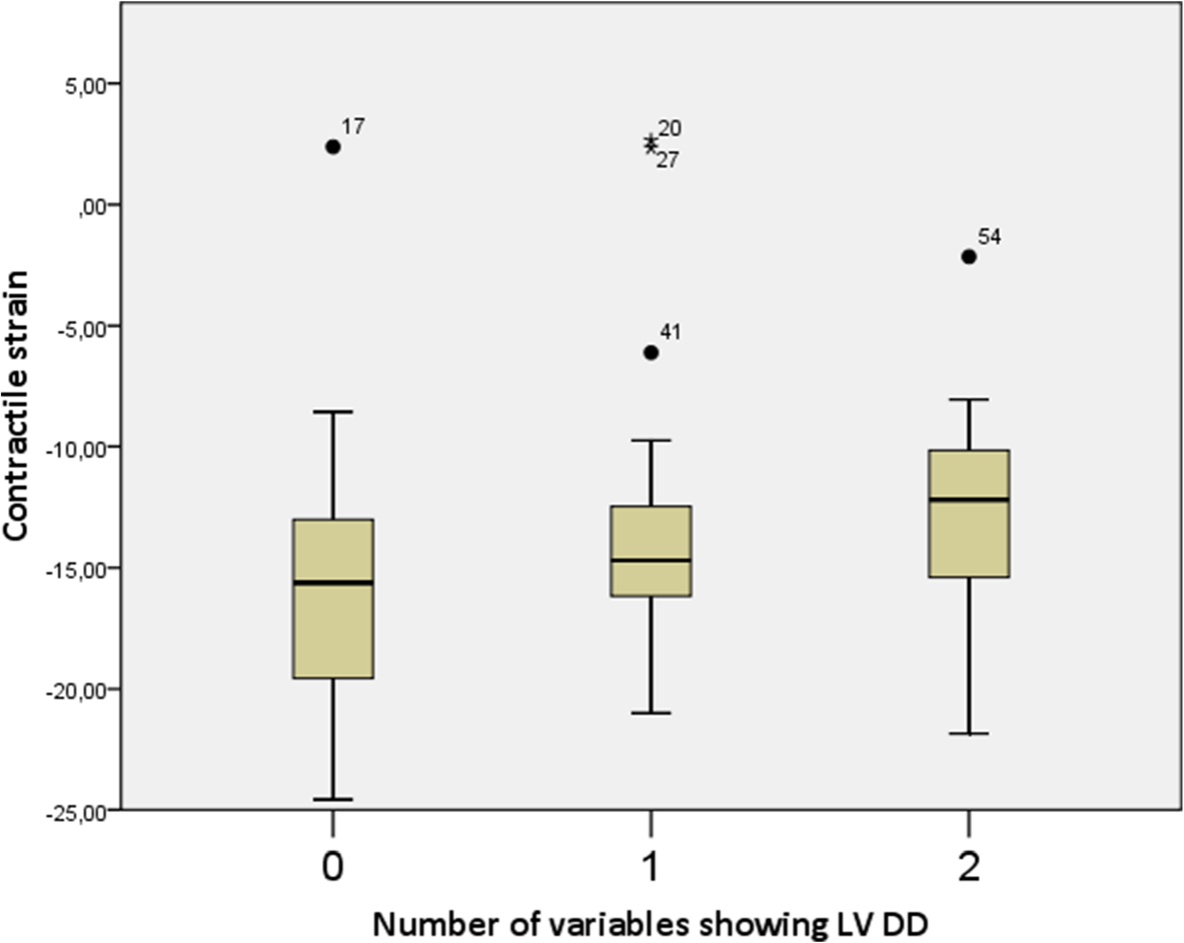
Contractile strain comparison between groups with different number of LV DD signs. There was no significant difference between the groups, *p* = 0.367

**Fig. 3:**
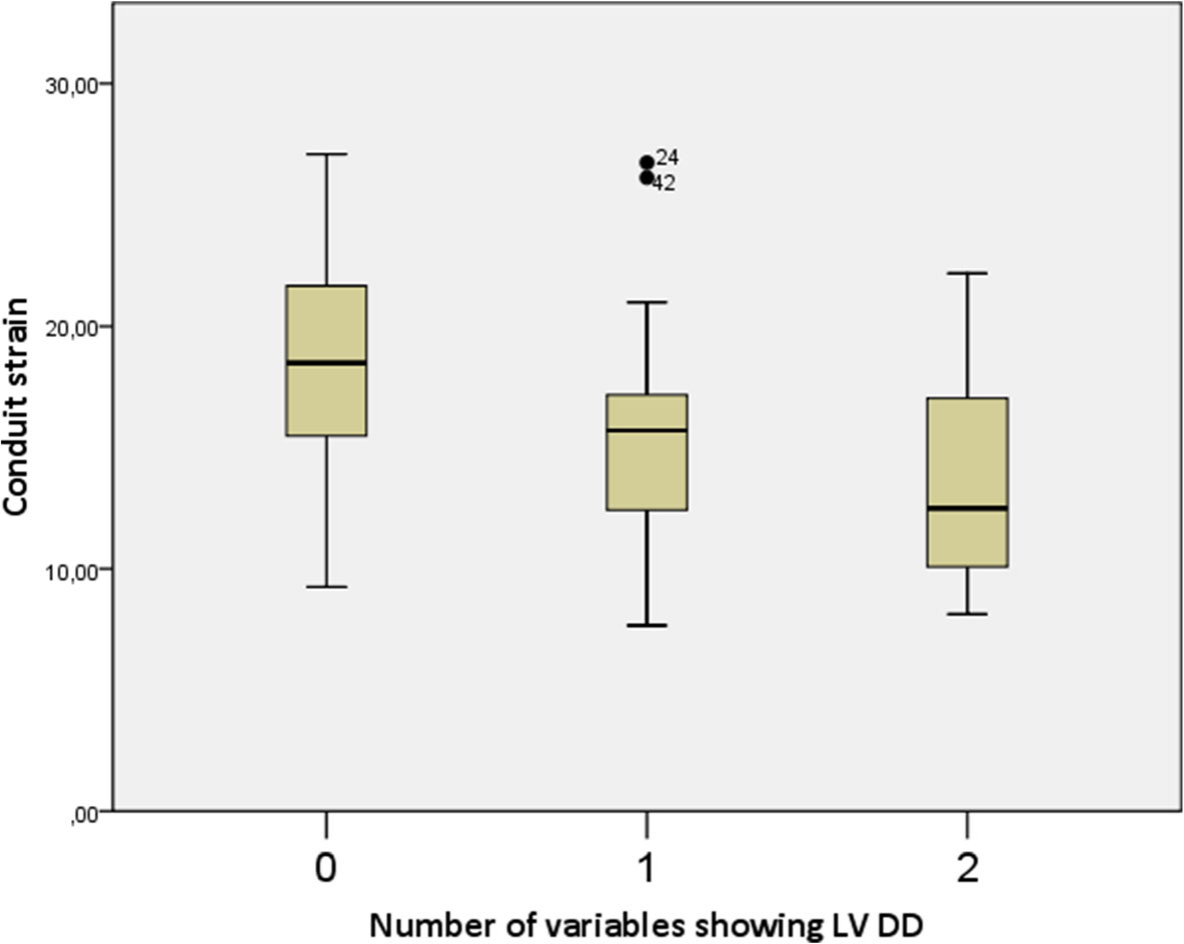
Conduit strain comparison between groups with different number of LV DD signs. Group with no signs of LV DD had higher conduit strain values (*p* = 0.016 vs 1 sign of LV DD and *p* = 0.001 vs 2 signs of LV DD). Difference between groups with 1 and 2 signs of LV DD were not statistically significant, *p* = 0.213

**Fig. 4:**
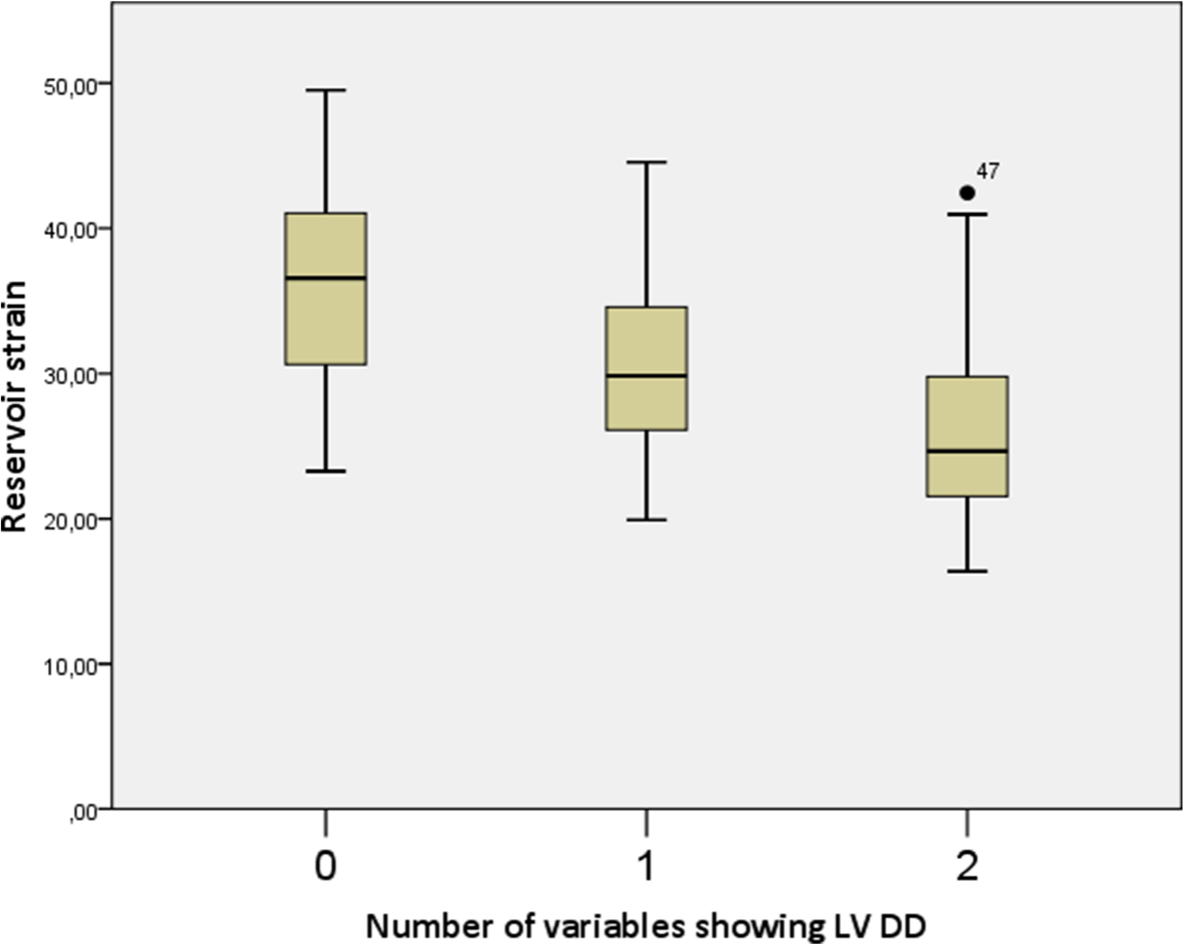
Reservoir strain comparison between groups with different number of LV DD signs. Group with no signs of LV DD had higher reservoir strain values (*p* = 0.014 vs 1 sign of LV DD and *p* < 0.001 vs 2 signs of LV DD). Difference between groups with 1 and 2 signs of LV DD did not meet the cutoff of significance, *p* = 0.072

**Fig. 5:**
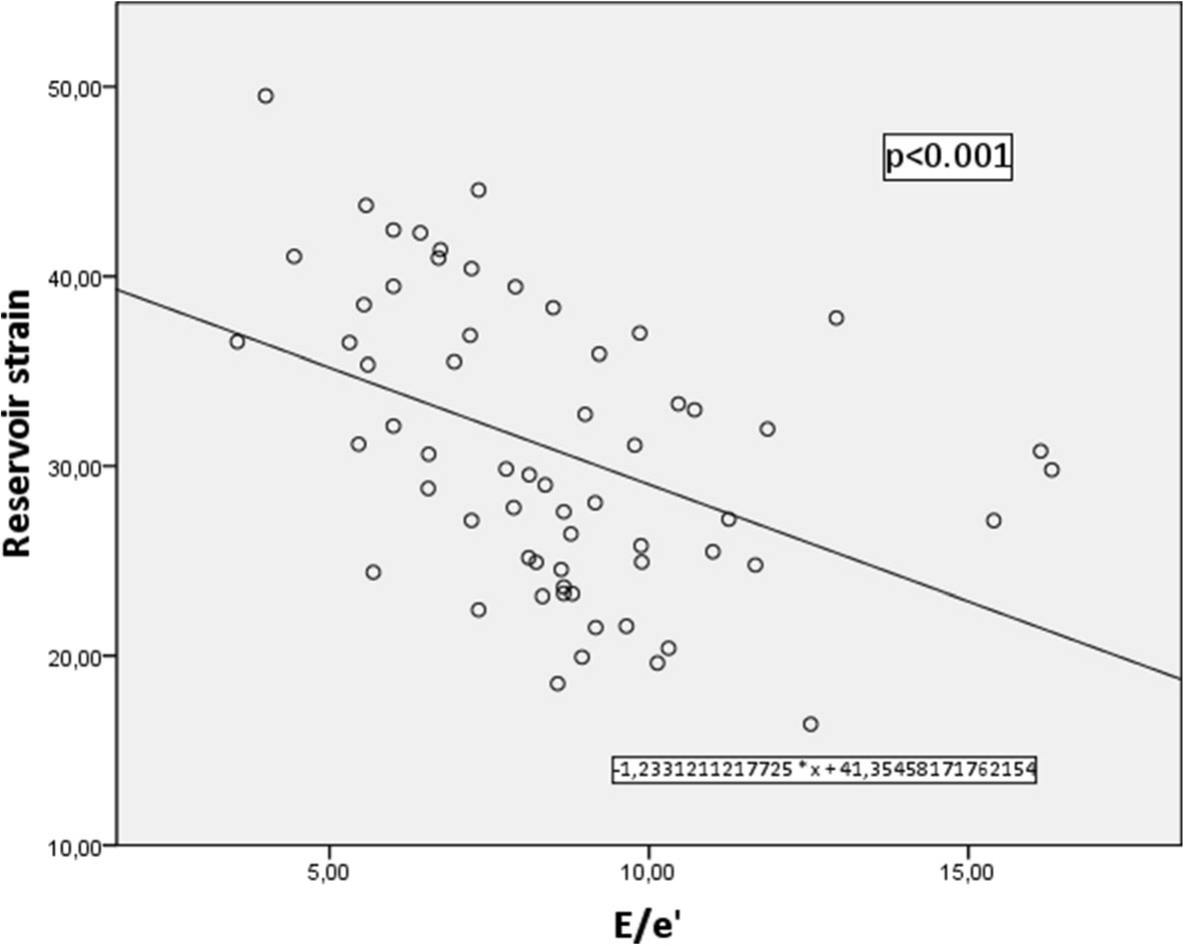
Scatterplot showing relation between reservoir strain and E/e’. There is significant correlation between marker of LV DD E/e’ and reservoir strain

Seventeen patients had no ultrasound signs of LV DD, 26 patients had one sign, 19 patients had two signs and only 1 patient had 3 ultrasound signs of LV DD, which allowed us to firmly diagnose LV DD according to the guidelines [12]. The single patient who had 3 signs of LV DD was excluded from further analysis, so we had three groups of patients with 0, 1 or 2 signs, showing LV DD. The mean contractile, conduit and reservoir strain values are shown in Table 5. Figures 2, 3 and 4 show graphical comparison of 2D LA strain data between these groups. Contractile strain differences between the groups were not statistically significant, *p* = 0.367. Conduit strain had statistically significant differences between groups. The group without any signs of LV DD had statistically significantly higher conduit strain (*p* = 0.016 vs 1 sign of LV DD and *p* = 0.001 vs 2 signs of LV DD). Reservoir strain also followed the same pattern as conduit strain with the group that had no signs of LV DD having statistically significantly higher reservoir strain values compared with other 2 groups (*p* = 0.014 vs 1 sign of LV DD and *p* < 0.001 vs 2 signs of LV DD). Reservoir strain difference between groups with 1 and 2 signs of LV DD did not meet the cutoff of significance, *p* = 0.072. We also checked for correlation of reservoir strain with average E/e’ ratio and found it to be statistically significant (p < 0.001) with correlation coefficient − 0.432. Regression analysis and scatter plot are shown in Fig. 5.

**Table 5:** LA strain values of patients with 0, 1, or 2 signs of LV DD

	Contractile strain mean (± SD)	Conduit strain mean (± SD)	Reservoir strain mean (± SD)
0 signs of LV DD	−15.68 (± 6.73) %	18.87 (± 4.44) %	35.82 (± 6.93) %
1 sign of LV DD	−13.74 (± 5.85) %	15.43 (± 4.57) %	30.48 (± 6.10) %
2 signs of LV DD	−12.96 (± 4.80) %	13.72 (± 4.30) %	26.68 (± 7.68) %

Strain values decrease as there are more signs of LV DD

## Discussion

The method of 2D strain imaging for the evaluation of LA function is being extensively studied and its role in risk determination is constantly increasing. Ability to maintain sinus rhythm after cardioversion or pulmonary vein isolation, reverse atrial remodeling after AF ablation, outcomes in patients with coronary artery disease, exercise capacity in heart failure, development of AF in valvular heart disease, even embolic complications in patients with AF – all these can be predicted by LA strain analysis [6, 18]. Though being so widely used and studied the method suffers from lack of standardization.

One of the main differences in the methodology is the reference point on the ECG. As most of the studies are done using GE software for LV strain analysis, the default setting is using ventricular cycle and zero reference point by default is set at the apex of R wave. Nevertheless, for the evaluation of atrial function using atrial cycle with zero reference point set at the start of P wave generates negative contractile function strain which is more “physiological” than positive value obtained with R wave reference point. There are more studies done with R being the reference point but most of the experts, including European taskforce members, agree that the onset of the P wave should be used to analyze LA strain in sinus rhythm as we did in our study [19–21].

Of no less importance is the question which parts of LA wall to include in the strain analyses. Expert consensus document of EACVI and EHRA on the role of multi-modality imaging for the evaluation of patients with AF recommends LA strain imaging to be performed only in the lateral wall [19]. This way the influence of nearby structures such as aorta and right atrium can be diminished. Despite that, there have been different approaches in multiple studies from the evaluation of all the segments in 4, 3 and 2 chamber apical views to just 6 segments in 4 chamber apical view. Though methodologically probably the correct approach would be to evaluate all the walls of the LA from 3 apical views [20, 21], the meta-analysis done by Faraz Pathan et al. revealed that the results of the studies using four-chamber view only, four- and two-chamber views, and four-, two-, and three-chamber views were very similar: 38% (95% CI, 35–41%), 41% (95% CI, 39–43%), and 39% (95% CI, 31–47%), respectively, and the difference was not statistically significant (*p* = 0.33) [8].

Our results show that all three LA functions are lower in patients with mild well treated arterial hypertension and paroxysmal AF compared with recently established normal values. These findings suggest that in hypertensive patients changes in the LA myocardium occur very early and support the role of LA strain imaging as an important and a very sensitive marker of hypertensive heart disease [22]. Recent data from Melissa Leung that links decreased LA reservoir strain with LA fibrosis, a fundamental component of hypertensive heart disease and AF, makes it even more valid and relevant in these conditions [23].

The second part of our analysis aimed to determine how LA strain parameters change with increasing number of parameters showing LV DD. The relation of LA strain parameters with different LV DD grades has been studied before and it seems that strain parameters follow a distinct pattern with the decreasing LV diastolic function. It has been shown that the most sensitive parameters of LV DD are reservoir and conduit strains, which significantly decrease even in mild LV DD and continue to decrease as the diastolic function gets worse. ROC curves show that diminished reservoir and conduit strains are superior even to LAVI in diagnosing early-stage LV DD. Meanwhile, contractile strain follows a different pattern. With mild LV DD LA contractility can even increase, dropping only when the DD is obvious [24, 25]. This seems logical and can be explained as reservoir and conduit strains mostly depend on LV longitudinal contraction and LA myocardial compliance whereas contractile strain is mostly influenced by LA myocardial contractility and LV filling pressures [26].

Our results confirm this strain changing pattern with decreasing LV diastolic function with the addition that the LA strain changes precede traditional signs of LV DD. Our patients were early in the course of developing DD as the majority of them had only 1 or 2 signs of LV DD which did not allow us to make an echocardiographic diagnosis of LV DD according to the guidelines [12] but they had already decreased LA strains. With increasing LV filling pressures and decreasing LV diastolic function reservoir and conduit strain values continuously decreased, while contractile strain values did not change or even increased, though it was not statistically significant.

If we followed the recently published guidelines on LV DD [12], only one patient in our study could be firmly diagnosed with LV DD. Significant part with decreased LA strains would fall into the indeterminate LV DD category. Probably this is the area where the LA strain parameters would be most helpful and could allow us to determine the risk of future cardiovascular events better.

## Conclusions

The patients with paroxysmal atrial fibrillation and primary arterial hypertension have decreased all 3 – reservoir, conduit and pump – functions assessed by 2D strain imaging even in the absence of echocardiographic signs of increased LV filling pressures. With increasing number of parameters showing high LV filling pressures LA conduit and reservoir functions decrease while contractile does not change or even increase. LA conduit and reservoir functions decrease earlier than the diagnosis of LV DD can be established according to the current guidelines in patients with primary arterial hypertension and AF.

## Abbreviations

AF: Atrial fibrillation
CI: Confidence intervals
DD: Diastolic dysfunction
EACVI: European Association of Cardiovascular Imaging
EHRA: European Heart Rhythm Association
LA: Left atrium
LV: Left ventricle
SD: Standard deviation
